# Studies of Intra-Fraction Prostate Motion During Stereotactic Irradiation in First Irradiation and Re-Irradiation

**DOI:** 10.3389/fonc.2021.690422

**Published:** 2021-07-14

**Authors:** Alexandre Taillez, Andre-Michel Bimbai, Thomas Lacornerie, Marie-Cecile Le Deley, Eric F. Lartigau, David Pasquier

**Affiliations:** ^1^ Academic Department of Radiation Oncology, Oscar Lambret Comprehensive Cancer Center, Lille, France; ^2^ University of Lille, Lille, France; ^3^ Department of Biostatistics, Oscar Lambret Comprehensive Cancer Center, Lille, France; ^4^ Department of Medical Physics, Oscar Lambret Comprehensive Cancer Center, Lille, France; ^5^ CRISTAL UMR CNRS 9189, University of Lille, Lille, France

**Keywords:** prostatic neoplasms, re-irradiation, stereotactic radiation therapy, motion, dose hypofractionation, salvage therapy, tracking

## Abstract

**Background:**

Understanding intra-fractional prostate motions is crucial for stereotactic body radiation therapy (SBRT). No studies have focused on the intra-fractional prostate motions during re-irradiation with SBRT. The objective was to evaluate these translational and rotational motions in primary treated patients and in the context of re-irradiation.

**Methods:**

From January 2011 to March 2020, 162 patients with histologically proven prostate cancer underwent prostate SBRT, including 58 as part of a re-irradiation treatment. We used the continuous coordinates of the fiducial markers collected by an orthogonal X-ray dual-image monitoring system. The translations and rotations of the prostate were calculated. Prostate deviations representing overall movement was defined as the length of the 3D-vectors.

**Results:**

A total of 858 data files were analyzed. The deviations over time in the group of primary treated patients were significantly larger than that of the group of re-irradiation, leading to a mean deviation of 2.73 mm (SD =1.00) versus 1.90 mm (SD =0.79), *P*<0.001. In the re-irradiation group, we identified displacements of -0.05 mm (SD =1.53), 0.20 mm (SD =1.46); and 0.42 mm (SD =1.24) in the left-right, superior-inferior and anterior-posterior planes. Overall, we observed increasing deviations over the first 30 min followed by a stabilization related to movements in the three translational axes.

**Conclusion:**

This is the first study to focus on intrafraction prostate motions in the context of re-irradiation. We observed that intra-fraction prostate motions persisted in the setting of re-irradiation, although they showed a significant reduction when compared with the first irradiation. These results will help to better estimate random errors during SBRT treatment of intra-prostatic recurrence after irradiation.

## Introduction

With an estimated 1.4 million new cases and 375,000 deaths worldwide, prostate cancer was the second most frequent cancer and the fifth leading cause of cancer death among men in 2020 (1). Radiation therapy has been validated as a standard treatment for localized prostate cancer (2, 3) and several radiation therapy methods have been developed. Studies have shown that by delivering high doses of radiation per session, stereotactic radiation therapy (SBRT) provides a control similar to that obtained with standard techniques (4–6).

An intra-prostatic recurrence is the site of first recurrence after normal fractionated radiation therapy (7). Traditional treatment options for the local treatment of intra-prostatic recurrence include radical prostatectomy, brachytherapy, cryotherapy, and high-intensity focused ultrasound (HIFU) (2). Re-irradiation using SBRT has emerged as an important technique for this indication showing, with a short follow-up of 26 months, a good local control rate of 83.2% (95% CI, 75.5% – 90.9%), a survival without biological recurrence of 59.3% (95% CI, 47.9% – 70.7%) with a low severe toxicity rate Grade ≥2 for gastrointestinal (GI) 1.1% (95% CI, 0.1% – 2.0%), and genitourinary (GU) 10.5% (95% CI, 5.5% –15.4%) (8–12).

Knowledge of the existence of intrafraction prostate motions during an extremely hypo-fractionated session is necessary to limit the volume already irradiated. The follow-up by X-ray orthogonal images of the Cyberknife^®^ (Accuray Incorporated, Sunnyvale, CA, USA) fiducial markers implanted in the prostate gland makes it possible to monitor the position of the target to take it into account when performing the treatment.

Several studies with a small number of patients focused on the intra-fractional prostate motions during the first stereotactic irradiation using the Cyberknife^®^. Their findings showed that the prostate underwent translational and rotational motions during a session (13, 14). However, to date, no studies have focused on prostate motions in the context of re-irradiation using SBRT. Therefore, this study aimed to investigate the intra-fractional prostate motions in the first irradiation and in three re-irradiations using SBRT with a Cyberknife^®^.

## Materials and Methods

### Screening of Patients

We collected the data from 162 patients treated at the Oscar Lambret Center (Lille, France), retrospectively. We included all the cancer patients treated with prostate SBRT using a dedicated Cyberknife^®^ VSI or Cyberknife^®^ M6 between January 1, 2011 and March 1, 2020.

The patients were divided into two different population groups, with the first group comprising patients with an indication for SBRT as a treatment for localized prostate disease who had never received local treatment, and the second group comprising patients treated with SBRT for an intra-prostatic recurrence after the first radiation of the external beam radiation therapy (EBRT) type or brachytherapy. Hormone therapy was administered before or during irradiation. Prostate biopsy was systematic before treatment initiation in both groups. With regard to the group of patients receiving re-irradiation with SBRT, we enrolled primary patients treated for prostate adenocarcinoma or other pelvic neoplasia. There was no rectal preservation strategy using an endorectal balloon or gel spacer. An empty rectum was used as the half-full bladder preparation protocol.

In the context of the first irradiation using SBRT, the prescription dose was 36.25 Gy in five fractions for an isodose of 80%. The clinical target volume (CTV) included the entire prostate gland and the proximal part of the seminal vesicles from patients classified as the intermediate-risk group according to the D’Amico classification. The margins of the planning target volume (PTV) were 5 mm in all directions, except in the posterior direction which was 3 mm. During focal or whole gland re-irradiation, the prescription dose was 36 Gy in six fractions for a prescription dose of 80%. The PTV margin was 2 mm (9).

### Acquisition of the Cyberknife^®^ Data

Two pairs of gold fiducial markers were placed in all the enrolled patients with the implantation of one pair at the apex and the other pair at the prostate base (15). To determine the position of the target when the patient was placed on the table, the data from the double orthogonal X-ray images taken at 45°and 135°in the horizontal plane and data from the digitally reconstructed radiograph (DRR), were reset. The readjustment was applied automatically on the treatment table.

The acquisition images of the fiducial marker follow-up were made automatically with the In-tempo^®^ system by adjusting the inter-image time according to the intra-fractional motions of the fiducial markers. In this system, the imaging and beam delivery was adapted to the rate and extent of tracked movements throughout the treatment, ensuring that accuracy is maintained from the first beam to the last. An automatic correction was then made to adjust the delivery of the beams (16). The deviations calculated from the radiographic images acquired in the time interval between the two motions of the table constituted a set of data.

The coordinates of the fiducial markers representing the prostate were collected throughout each session (with a median time of 50 s between two images) with treatment information for each beam, the beam and node number, and the movement of the target position.

### Statistical Analyses

In each session with each patient, we analyzed the motions in relation to the reference point defined at the start of the session which corresponded to the barycenter of the fiducials after the first follow-up image.

The coordinates were recorded in three planes to measure the lateral, vertical, and longitudinal motions: “LR (Left-Right),” “SI (Superior-Inferior)” and “AP (Anterior-Posterior). Rotational motions were also recorded (“Roll,” “Pitch,” and “Yaw”). At each measurement time, we calculated the deviation from the reference point as the square root of the sum of the squares of the measurements “LR (Left-Right),” “SI (Superior-Inferior)” and “AP (Anterior-Posterior).” This deviation represented the overall prostate motion (length of the 3D vector).

For each session, we estimated the area under the deviation curve (AUC) for all treatment times up to 60 min; measurements after 60 min were ignored because of the low number of fractions that lasted more than 60 min. We then estimated the mean deviation for each session by dividing the AUC by the session’s treatment time (shortened to 60 min). The mean deviation was estimated per patient to compare the treatment groups (primary irradiation versus re-irradiation) using the Student’s t-test.

The deviation time variations were described considering the distribution of this parameter by 10-minute time interval, between 0 and 60 minutes, overall and by treatment group (primary irradiation vs. re-irradiation).

The deviation was modeled using a mixed linear regression which made it possible to estimate the mean difference between the two treatment groups. This took into account the time effect, overall, and according to treatment group (time × treatment interaction) while considering the patient factor as a random factor. With regard to the six basic measurements of motion “LR (Left-Right)”, “SI (Superior-Inferior)”, “AP (Anterior-Posterior)”, “Roll”, “Pitch” and “Yaw”, we calculated their means and standard deviations for each 10-minute time interval [(0,10), (10, 20)…(50-60)].

The significance of the test was set at *P*<0.05. All the statistical analyses were performed using STATA v15.

## Results

### Description of Populations

After excluding five patients who objected to the use of their medical data, the study population consisted of 162 patients whose median age at enrollment was 73 years old. Among the 162 patients, 58 (35.8%) received stereotactic re-irradiation, and 104 received their first stereotactic radiation (64.2%). A total of 858 sessions were analyzed. The patient and tumor characteristics during SBRT treatment are described in **Table 1**.

**Table 1:** Patient and tumor characteristics at the time of SBRT (N=162).

Characteristics	1st irradiation N=104		Re-irradiation N=58		Total N =162	
**Age (years)**						
Median (min.; max.)	75	(54 – 85)	70	(51 – 87)	73	(51 – 87)
**ECOG Performance Status**	(M=4)					
0	77	77.0%	52	89.7%	129	81.6%
1	22	22.0%	6	10.3%	28	17.7%
2	1	1.0%	0	0.0%	1	0.6%
**History of pelvic surgery**						
No	103	99.0%	52	89.6%	155	95.7%
Yes	1	1.0%	6	10.3%	7	4.3%
**PSA (ng/mL)**						
Median (min.; max.)	8	(2.3 – 78.0)	5	(0.4 – 39.0)	7	(0.4 – 78.0)
**Gleason score**			(M=7)		(M=7)	
≤ 6	48	46.2%	7	13.7%	55	35.5%
3+4	40	38.5%	8	15.7%	48	30.1%
4+3	12	11.5%	8	15.7%	20	12.9%
≥ 8	4	3.9%	22	43.1%	26	16.8%
N/A¹	0	0%	6	11.8%	6	4.9%
**Prognostic group of Amico**						
Favorable	35	33.7%				
Favorable intermediate	40	38.5%				
Unfavorable intermediate	17	16.3%				
High risk	12	11.5%				

M, missing data; N/A¹, anatomical pathology analysis not feasible; ECOG, Eastern Cooperative Oncology Group; PSA, prostate-specific antigen.

The initial characteristics of the patients who received SBRT after re-irradiation are described in **Table 2**. Among these 58 patients, 49 (84.5%) received the first irradiation for prostate neoplasia. Six re-irradiations were performed after the neoadjuvant treatment of rectal cancer and three after other indications (lymph nodes metastases of cutaneous neuroendocrine carcinoma, bladder urothelial carcinoma, and retroperitoneal liposarcoma). Three-dimensional conformal radiation therapy was the initial technique that was used, with 69% of the irradiation being in the context of a first indication. Prostate brachytherapy was performed in 14 patients (24.1%). Previously irradiated prostate disease was most often confined to the prostate gland (75.5% classified as cT1 and cT2).

**Table 2:** Patient, tumor and treatments characteristics at the time of first irradiation in patients who had SBRT as “re-irradiation” (N=58).

Characteristics		
**Neoplasia related to 1st irradiation**		
Prostate	49	84.5%
Rectum	6	10.3%
Other	3	5.2%
**Technique used during the 1st irradiation**		
IMRT	4	6.9%
3D-CRT	40	69.0%
Brachytherapy	14	24.1%
**Abdominal-pelvic amputation**		
Yes	0	0%
No	58	100%
**Dose of the first radiation (Gy)**		
Median (min.; max.)	70.1	(45 – 78)
**D’AMICO prognostic group during the 1st irradiation (N=49)**		(M=2)
Favorable	16	34.0%
Favorable intermediate	8	17.0%
Unfavorable intermediate	2	4.3%
High risk	21	44.7%
**TNM-staging of prostate cancer** **for a first irradiation (N=49)**		(M=4)
cT1a	1	2.2%
cT1b	1	2.2%
cT1c	14	31.1%
cT2a	10	22.2%
cT2b	3	6.7%
cT2c	5	11.1%
cT3a	6	13.3%
cT3b	3	6.7%
cT3aN1	1	2.2%
cT3bN1	1	2.2%

M, missing data; IMRT, intensity-modulated radiation therapy; 3D-CRT, three-dimensional conformal radiation therapy.

### Duration of Treatments

With regard to the duration of the sessions, they lasted on average 42.2 minutes ( ± 12.5) for primary irradiation and 40 minutes ( ± 17.3) for re-irradiation. Less than 10% of the sessions lasted more than 60 minutes (80/858). As shown in **Figure S1 (Appendix)**, most sessions lasted between 30 and 50 minutes (243 sessions, 28.3% between 30 and 40 minutes, and 234 sessions, 27.3%, between 40 and 50 minutes).

### Description of Motions

**Figure 1:**
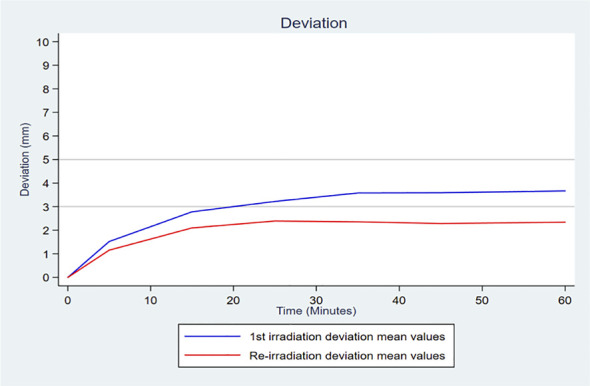
Curve of the mean deviation per 10-minute interval in each treatment group (primary irradiation and re-irradiation).

**Figure 1** describes the changes in the deviations over time according to the treatment group (primary irradiation and re-irradiation). The mean deviation over time in the primary irradiation group was significantly greater than that in the re-irradiation group (mean deviation of 2.73, SD =1.00, *versus* 1.90, SD =0.79, respectively, *P*<0.001), demonstrating an increased prostate mobility for primary irradiations.

The result of the mixed linear regression confirmed a significant temporal trend (*P*<10-4) and significant mean differences between the two groups, estimated at -0.71 mm (95% CI, -1.01 to -0.40; *P*<10-4) when the model was adjusted only over time. The model with interaction made it possible to conclude that not only was there was a significantly different mean deviation between the two groups, there was also a greater increase in the deviation over time in the primary irradiation group than in the re-irradiation group (the gradient being 0.51 mm and 0.43 mm for 10 minutes of time respectively, with a significant time x treatment interaction test, *P*<10-4) (**Table A1 in the Appendix**).

With regard to the variability over time of the prostate motion around the average, the results showed that motions of re-irradiation were -0.05 mm (SD = 1.53) for the LR translation, -0.2 mm (SD =1,46) for the SI translation, and 0.42 mm (SD = 1.24) for the AP translation.

Concerning the temporal evolution of the prostate motions on the rotational axes in re-irradiation, it is noted that these motions remained close to the position observed at the beginning of the session, particularly for the roll (average = 0.02°, SD = 0.81°) and yaw (average = 0.05°, SD = 0.65°) axes. On the pitch, we observed a rotational average of − -0.13° with a SD of 1.52°

**Figure 2:**
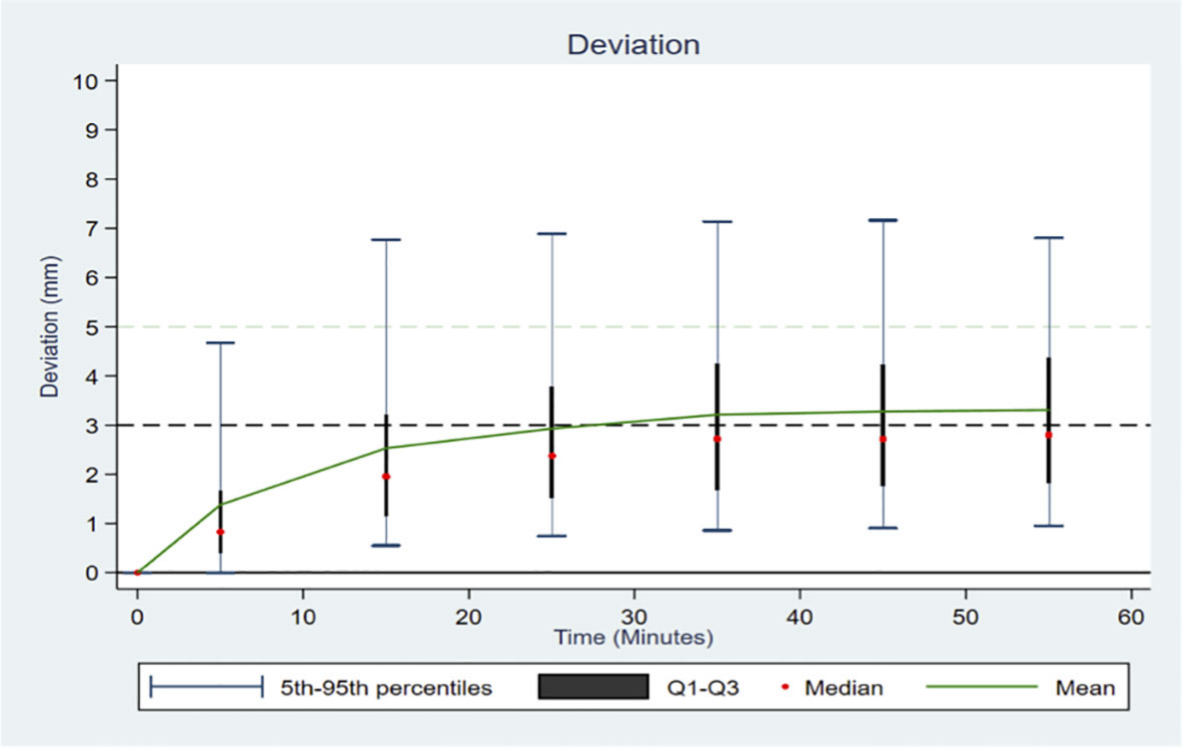
Distribution of the deviation according to time in 10-minute intervals, across all patients.

**Figure 2** shows the changes in the deviations over time for the entire study population. Considering the 10-minute time intervals, there was an increase in the deviations over the first 30 minutes (median of 0.82, 1.94 and 2.37 mm in the intervals 0 – 10, 10 – 20, and 20 – 30, respectively) with a stabilization of the deviation after the first 30 minutes (median of 2.74, 2.75 and 2.82 mm in the intervals 30 – 40, 40 – 50, and 50 – 60, respectively). In the time intervals after the first 20 min, more than 35% of the recorded deviation values were measurements above 3 mm, and more than 14% were above 5 mm (**Figure S2 in the appendix**).

**Figure 3:**
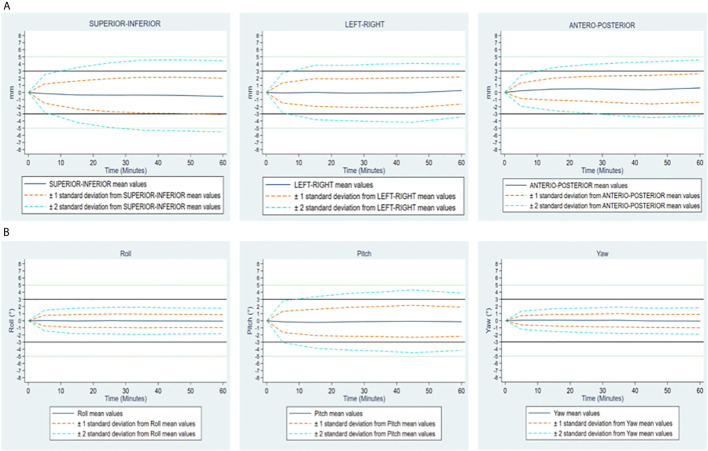
**(A)** Translational motions Supero-Inferior, left-Right and antero-posterior expressed in mm. **(B)** Rational motions Roll, Pitch and Yaw expressed in degrees. Changes over time of translational and rotational motions of the prostate, in all patients. On each figure, mean values and standard deviations, by 10-minute time interval, overall considering both groups together.

**Figure 3** illustrates the mean motions and dispersion of these motions over time for all the sessions and patients. We observe more translational motions (for all the measurements, SD = 2.05, 1.86 and 1.60 mm for the LR, SI and AP translational motions respectively) and “Pitch” rotations (SD = 1.86°), contrasting with a low variability in “Roll” and “Yaw” rotations (SD = 0.88 and 0.81° respectively). The histogram of the distribution of the different measurements is illustrated by 20-minute intervals (0 – 20, 20 – 40, and 40 – 60) in **Appendix Figure S3**.

## Discussion

The delivery of a large number of small, non-isocentric, and non-coplanar beams directed at a target with a sub millimetric precision near the organs at risk, requires knowledge of prostate motions, especially since they are random and unpredictable (17). Our data suggested that during the first stereotactic irradiation of the prostate and during stereotactic re-irradiation after another radiation therapy technique, there were small but significant differences in the intra-fractional prostate motions.

To our knowledge, this is the first study to analyze intra-fractional prostate motions during stereotactic re-irradiation. This is a retrospective study but all treated patients have been included and we used technical data, so the retrospective nature does not influence the results.

One of the hypotheses for the weakest intra-fractional prostate motion is the onset of pelvic fibrosis following the first irradiation. Another hypothesis is better knowledge of preparation instructions during re-irradiation, since the patient had already applied them previously. Indeed, patients with experience in long external radiation therapy (with almost 40 fractions) could be able to better apply preparations instructions when starting a new irradiation.

The extent of intra-fractional motions is disputed. Some studies that focused on the motions during a shorter irradiation with intensity-modulated radiation therapy (IMRT) have reported a significant number of necessary corrections, while others have described only more insignificant motions. These studies used different imaging systems as tools, such as the megavolt (18), megavolt-kilovolt imaging (19), Varian Calypso System (Varian Medical Systems, Palo Alto, CA,USA) (20, 21), and magnetic resonance imaging (MRI) (22).

With an increase in treatment duration, the significance of intra-fractional motion has grown, with appreciable variation being demonstrated. For the first 10 minutes of traditional radiation therapy, observations are similar to the multiple data that can be found in the literature focusing on prostatic motion.

Real-time tracking methods using orthogonal kV X-ray imaging with Exatrac Optical System showed average intra-fractional motion (± 1 SD) in the LR, SI, and AP directions of 0.7 ± 0.5 mm, 1.3 ± 0.7 mm, and 1.4 ± 0.9 mm respectively (23). Other studies such as Willoughby et al. have used an electromagnetic tracking system with Calypso^®^ for prostate real-time tracking during external beam radiotherapy and their results showed that the average (SD) of the maximum differences were 0.91 ± 0.35 mm, 3.61 ± 3.13 mm, and 3.92 ± 4.32 mm in the lateral, longitudinal, vertical directions, respectively (24). Motion can also be studied with MRI. For instance, Mah D et al. showed prostate displacements (mean ± SD) of: 0.2 ± 2.9 mm, 0.0 ± 3.4 mm, and 0.0 ± 1.5 mm in the anterior–posterior, superior–inferior, and left-right dimensions respectively (25).

The increase of motion with time has also been demonstrated in conventional fractionation by IMRT (26). For example, a study using a total of 68 sagittal cine-MRI sequences demonstrated an increasing displacement in the AP and SI planes during treatment with SD of 0.57 mm and 0.41 mm in the first two minutes increasing to 1.44 mm and 0.91 mm in the two to four minutes. This appears to be consistent with the increase in motion over time found in our study (27).

With the Cyberknife^®^, since the treatment time was close to 40 min per session, tracking was considered to be the most suitable solution. There is a tendency for more extensive motions when the session is long (17, 28, 29). Classic linear accelerators also allow stereotactic prostate radiotherapy to be performed. The treatment time is much shorter and image-guided radiation therapy (IGRT) techniques are different.

With regard to the translational components LR, SI, and AP during the first stereotactic irradiation by Cyberknife^®^, compared to the results of previous studies, our results were homogenous. Moreover, Koike et al. (30), based on the files of 16 patients, reported an LR of -0.09 ± 0.81 mm, a SI of 0.15 ± 2.06 mm, and an AP of 0.79 ± 1.99 mm, as well as an average deviation of 2.53 ± 1.77 mm. Similarly, Choi et al. (14), with data from 71 patients, found the translational averages for LR to be 0.12 ± 0.19 mm, SI 0.15 ± 0.31 mm, and AP 0.73 ± 0.32 mm with an average deviation of 1.0 ± 0.35 mm. Furthermore, Xie et al. (13) used data from 21 patients and found that for the LR, SI, and AP directions, values were 0.87 ± 1.17 mm 1.55 ± 1.28 mm, and 1.80 ± 1.44 mm, respectively. Our average deviation data were consistent with the results of Xie et al. (13), showing a deviation of 2.61 mm ( ± 1.94 mm) during *de novo* irradiation. With regard to rotational prostate motions, in the work of Wolf et al. (31), the rotational data of 20 patients were evaluated, showing pitch rotations of 3.6° (SD 4.9°), roll 0.2° (SD 2.1°) and yaw 0.1° (SD 2.1°). The analysis by Cuccia et al. (32) showed rotations of the yaw at 0.09 ± 0.10°, pitch -0.04 ± 0.33°, and roll 0.18 ± 0.15°.

Other analyses of prostate motions were presented more recently as part of an irradiation with a magnetic resonance imaging-guided linear accelerator (MRI-LINAC), where the time per fraction was quite close to that performed with the Cyberknife^®^, that is, between 30 and 50 min per session (32, 33). Data from Cuccia et al. (32) on 100 fractions showed translational motions such as LR -0.24 ± 2.5 mm, SI 0.06 ± 0.46 mm and AP -0.17 ± 0.91 mm.

Our study found mainly translational motions in AP and SI, as observed by Langen et al. (28, 34), and there was a continuously increasing motion independent of the first irradiation or re-irradiation group, in line with the findings of other studies using prostate coordinates during irradiation by MRI-LINAC, particularly with respect to the findings of Keizer et al. (33).

The addition of a rectal preservation strategy has also been studied in the context of irradiation with SBRT. In other words, Cuccia et al. (32) were interested in the influence of the hydrogel spacer on the intra-fraction motions during irradiation with MRI-LINAC, and it was reported that the pitch rotation decreased significantly due to the use of this strategy. The use of the endorectal balloon or hydrogel spacer in SBRT is a possible option that has shown benefits, particularly in dosimetry (35, 36).

SBRT salvage therapy has been evaluated mainly retrospectively (8) and several prospective multicenter studies are ongoing (11, 12, 37)

Our study did not investigate the causes that could influence prostatic movements during a session, although displacements greater than 5mm were observed in 14% of patients. However, several investigators have shown that non-resolving slow drift, mainly in the AP direction, is due to rectal filling, and that sudden transient motion, most frequent in AP and SI directions, is due to intestinal peristalsis. These are the two main types of prostate motion during a session. Pelvic muscle contraction can also contribute to AP plan. Systematic and random motions are significant in the AP and SI axes, while they are less significant in the LR axis (26, 38).

In our study, re-irradiation was the only factor that influenced prostate motion.

Several stereotactic radiation therapies exist in clinical routine and there are many IGRT methods. Image tracking with InTempo^®^, Exatrac^®^ (ExacTrac, BrainLAB AG, Heimstetten, Germany) or transponders such as Calyspo^®^ (Varian Medical Systems, Palo Alto, CA) are another way to track intra-fractional motion of a target. Real-time image tracking is all the more significant if the treatment time is long since we know that the movements can be more important (27, 39).

Currently, the only truly real-time IGRT methods are presented by MRI-Linac and Calypso^®^ monitoring, however their accessibility is low worldwide. One of the strengths of the Cyberknife is that it can adapt the time between each image according to the motions previously recorded.It is therefore an adaptive discontinuous tracking almost in real time (Kv imaging between 15 and 150 seconds). Using Linac, a cone-beam-CT/Kilovoltage (Kv) follow-up can estimate the intra-fraction prostatic position between each arc but cannot be used during treatment delivery to assess for intrafraction organ motion especially because of prostate abrupt movements.

Some stereotactic irradiations are performed without real time tracking and we believe that in the context of a re-irradiation, real time tracking should be privileged although its clinical relevance is not established.

Finally, Choi et al. (14) showed that prostatic motion in the AP plane and global deviation had a possible association with digestive and urinary toxicities during Cyberknife^®^ SBRT despite automatic correction. It therefore appears relevant to better understand prostatic motion in a context of increased risk of toxicity due to re-irradiation in order to better argue the practical management of the treatment.

The practices with regard to the implementation of the PTV in the context of re-irradiation with SBRT differ, being 0 mm in the study by Fuller et al. (11), 3 mm for Bergamin et al. (12), and 2 mm for Pasquier et al. GETUG 31 (37).

Reducing PTV margin is crucial since the reduction of the planned volume leads to less exposure to toxicity for organs at risk (40). PTV margin creates a fictitious volume that provides an acceptable probability of the delivery of CTV or GTV prescription dose. Although it is complex to calculate PTV margin in stereotactic radiotherapy, we can confirm that intra-factional motions are essential for its estimation (41).

Since we observed less motion during re-irradiation its seems relevant to use a smaller margin compared to the margins used in first irradiation, especially since organs at risk are subject to strict constraints, dose gradient is high and the number of fraction is limited.

## Conclusion

This study analyzed intra-fractional prostate motions during stereotactic irradiation as the first treatment and re-irradiation. Intra-fraction prostate motions persisted in the setting of re-irradiation, although a significant reduction was observed when compared to the first irradiation. The findings of our study make it possible to better understand prostate behavior at a time where re-irradiation by SBRT is being evaluated as a salvage therapy for intra-prostatic recurrence.

## Data Availability Statement

The raw data supporting the conclusions of this article will be made available by the authors, without undue reservation.

## Ethics Statement

Ethical review and approval was not required for the study on human participants in accordance with the local legislation and institutional requirements. The patients/participants provided their written informed consent to participate in this study.

## Author Contributions

Conceptualization, DP and AT. Methodology, M-CLD, DP and AT. Formal analysis, A-MB. Investigation, AT and DP. Resources, TL and EFL. Writing—original draft preparation, AT. Writing—review and editing, DP, TL, and M-CLD. Supervision, DP and AT. Project administration, AT. All authors contributed to the article and approved the submitted version.

## Conflict of Interest

The authors declare that the research was conducted in the absence of any commercial or financial relationships that could be construed as a potential conflict of interest.

## Abbreviations

SBRT, stereotactic body radiation therapy; SD, standard derivation; HIFU, high-intensity focused ultrasound; EBRT, External Beam Radiation Therapy; GTV, Gross Tumor Volume; CTV, Clinical Target Volume; PTV, Planning Target Volume, DRR, Digitally Reconstructed Radiograph; LR, Left-Right; SI, Superior-Inferior, AP, Anterior-Posterior; AUC, area under the deviation curve; Kv, Kilovoltage; IMRT, intensity-modulated radiation therapy; MRI-magnetic resonance imaging; IGRT, image-guided radiation therapy; MRI-LINAC, magnetic resonance imaging-guided linear accelerator.
