# Supplementary material for: Studies of Intra-Fraction Prostate Motion During Stereotactic Irradiation in First Irradiation and Re-Irradiation
Source: Front Oncol. 2021 Jul 14;11:690422. doi: 10.3389/fonc.2021.690422 (PMC8316636; doi:10.3389/fonc.2021.690422)
Supplement: Supplementary file 1 [file Table_1.docx]

Supplementary Material

# Supplementary Table

**Table S1:** Results of the mixed linear regression of the deviation

|  | **Model without interaction term** | | | **Model with an interaction between group and time** | | | | |
| --- | --- | --- | --- | --- | --- | --- | --- | --- |
|  | Coefficient  (mm) | IC95%  (mm) | P value |  | Coefficient  (mm) | IC95%  (mm) | P value | P-value interaction |
| - Group (re-irradiation compared to first irradiation) | -0.71 | (-1.01 ; -0.40) | <10-4 | - Group(re-irradiation compared to first irradiation) | -0.53 | (-0.84 ; -0.22) | 0.001 |  |
| - Time - (/10 min) | 0.49 | (0.47; 0.50) | <10-4 | - Time (/10 min)  in the group of  1st irradiation | 0.51 | (0.49; 0.53) | <10-4 | <10-4 |
|  |  |  |  | - Time (/10 min)  in the group of  re-irradiation | 0.43 | (0.40; 0.45) | <10-4 |  |

Results of the model with the “time x group” interaction term are presented using contrasts, leading to the estimate of time effect (slope of linear regression) separately in both groups. The difference of slopes between both groups is tested by the interaction test.
