# Supplementary figures and images for: Studies of Intra-Fraction Prostate Motion During Stereotactic Irradiation in First Irradiation and Re-Irradiation
Source: Front Oncol. 2021 Jul 14;11:690422. doi: 10.3389/fonc.2021.690422 (PMC8316636; doi:10.3389/fonc.2021.690422)

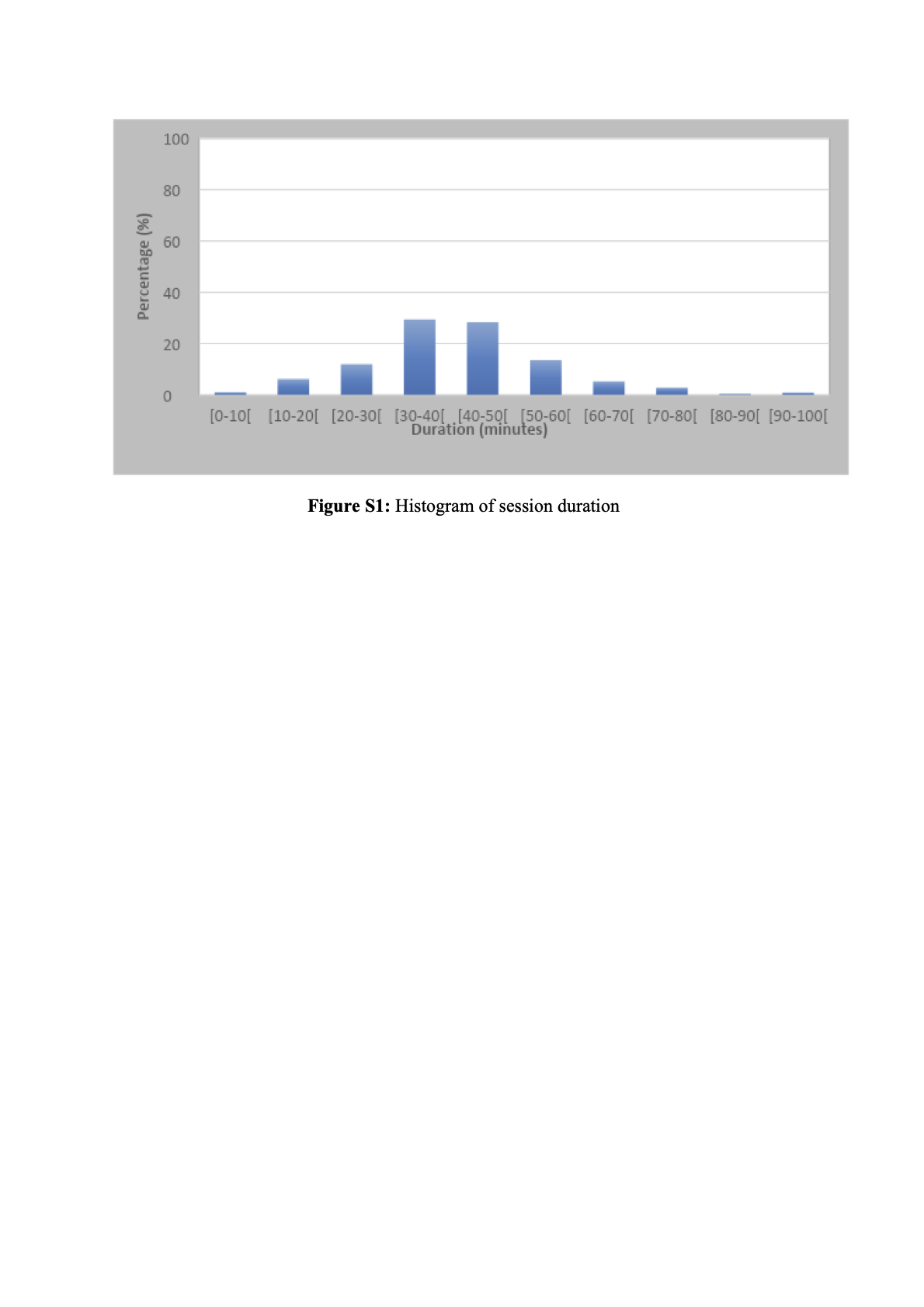

Supplement: Supplementary file 2 [file Image_1.tiff]

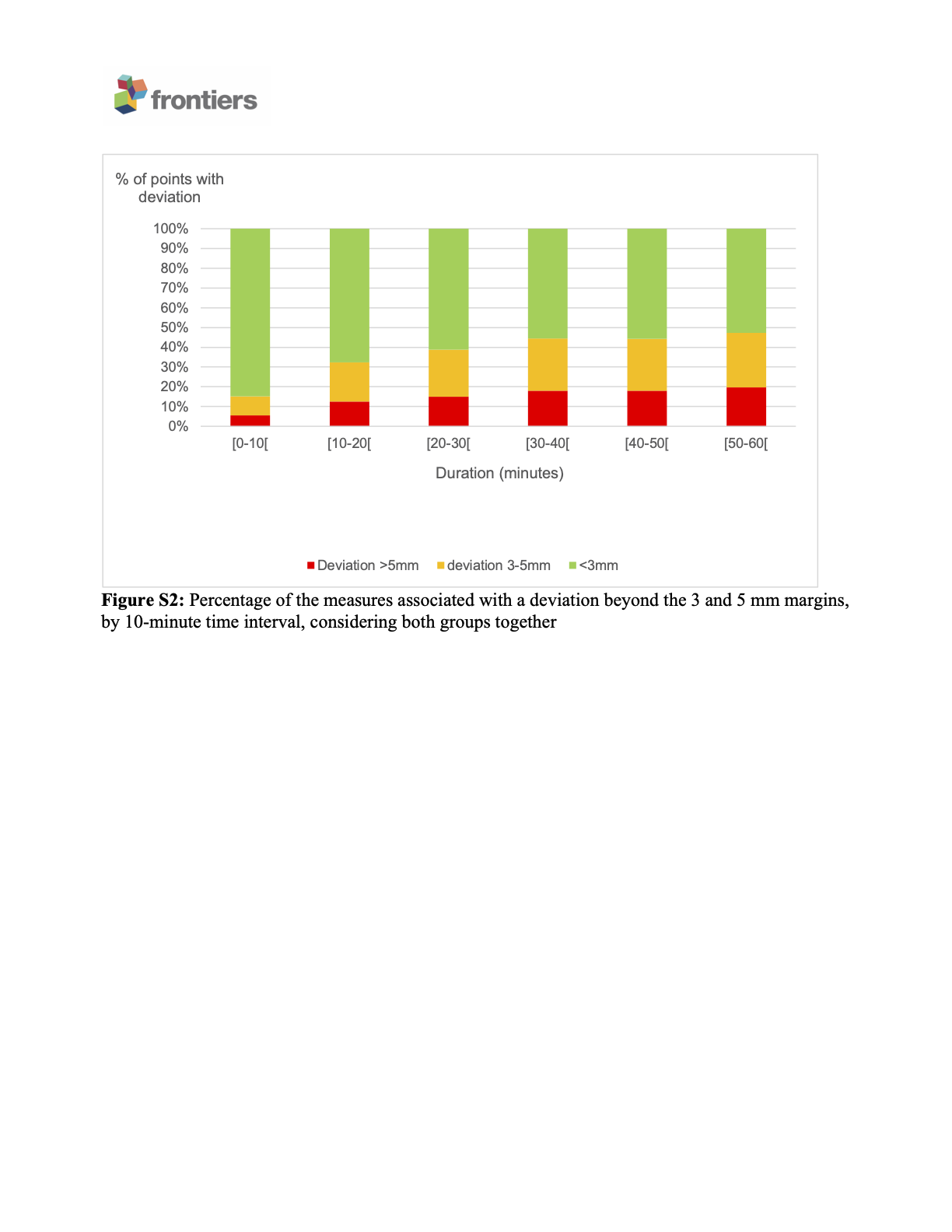

Supplement: Supplementary file 3 [file Image_2.tiff]

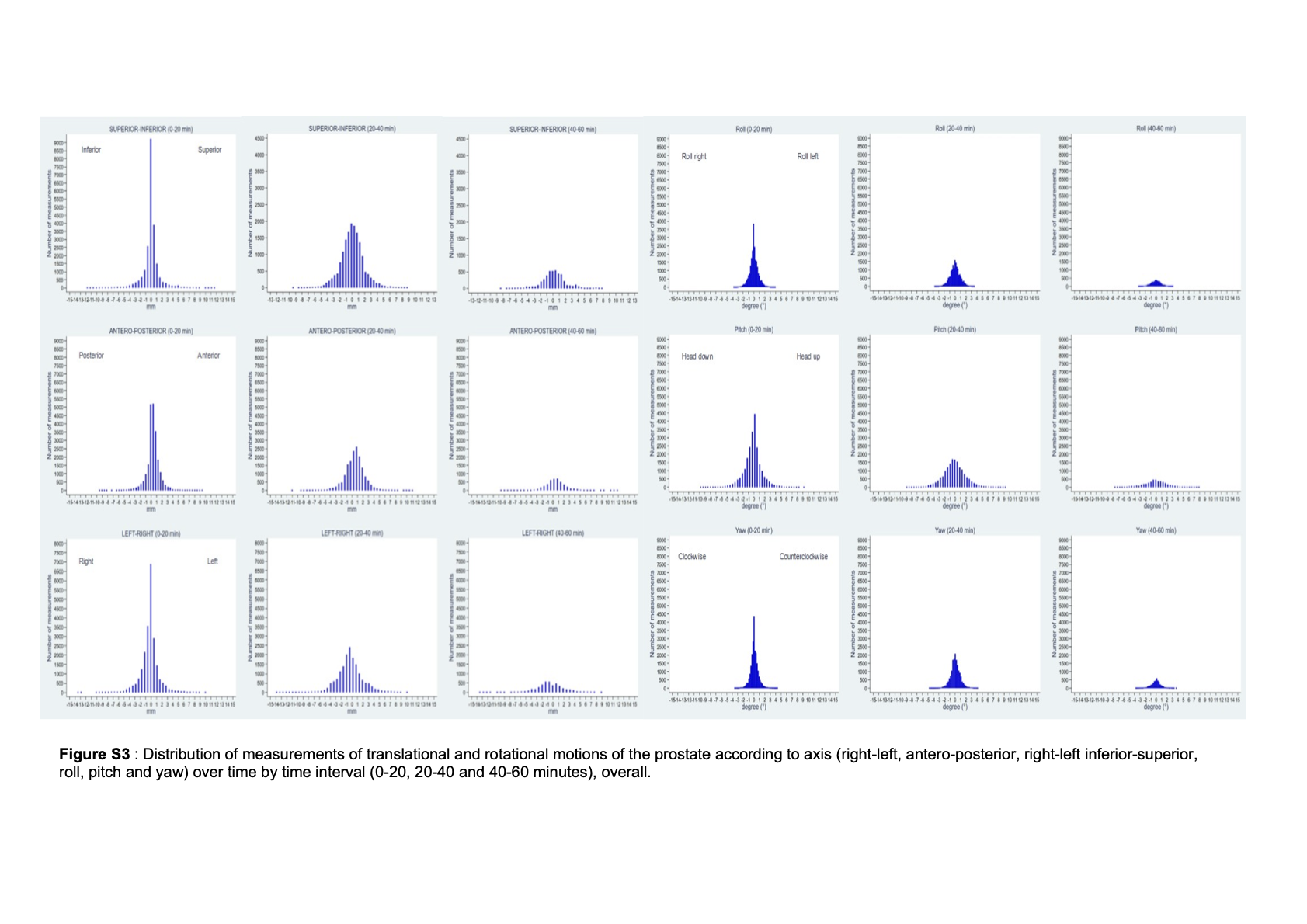

Supplement: Supplementary file 4 [file Image_3.tiff]
